# Evaluating Adherence to the National Institute for Health and Care Excellence (NICE) Guidelines in the Management of First-Presentation Low Back Pain: A Retrospective Audit in Primary Care

**DOI:** 10.7759/cureus.97116

**Published:** 2025-11-17

**Authors:** Alisa Khalid, Rayyan Khalid

**Affiliations:** 1 Trauma and Orthopaedics, University Hospital Crosshouse, Kilmarnock, GBR; 2 Medicine, University of Glasgow, Glasgow, GBR; 3 Medicine, The University of Edinburgh, Edinburgh, GBR

**Keywords:** lower back pain (lbp), nice guidelines, patient education, patient health education, physician guideline adherence, primary medical care, retrospective audit

## Abstract

Introduction

Low back pain (LBP) is one of the most common presentations in primary care and a leading cause of disability worldwide. The global prevalence of LBP continues to rise, with an increasing burden on healthcare systems. Adherence to clinical guidelines is essential to optimise management and avoid unnecessary investigations. This audit aimed to assess adherence to the National Institute for Health and Care Excellence (NICE) guidelines in the management of first-presentation LBP in primary care.

Methods

This retrospective audit was conducted at a single general practice in Glasgow, Scotland. Data were collected from a random sample of 71 patients with their first episode of LBP and no red-flag symptoms between January 2024 and February 2025. Current practice was compared with the NICE guidelines for the management of first-presentation LBP. Patient selection was performed using a computer-generated random-number function in Microsoft Excel (Microsoft Corporation, Redmond, Washington) to minimise selection bias and ensure that the findings reflected typical patterns of LBP management within the practice population. Adherence to guidelines was assessed across key domains, including patient education, self-management advice, pharmacological treatment, and physiotherapy referral.

Results

The audit found that, overall, 39.4% (n = 28) of cases were fully compliant with the recommended NICE guidelines. Patient education was the most compliant method, which commonly included self-management advice regarding weight loss and regular exercise. Non-steroidal anti-inflammatory drugs were prescribed in 21.1% of cases, and opioids were prescribed in 9.9%, opposed to guideline advice. Physiotherapy self-referral occurred in 39.4% of cases, and fit notes for work absence were issued in 4.2%. These findings highlight a variation in clinical practice and areas to improve compliance with the NICE guidelines.

Conclusion

The audit identified significant gaps in adherence to the NICE guidelines in the management of first-presentation LBP in primary healthcare. Following the audit, a poster summarising the NICE guidelines was created and presented at a departmental meeting to promote staff awareness. This targeted education and feedback may improve compliance with evidence-based management of first-presentation LBP in primary care. A re-audit is planned to evaluate the improvement following these measures.

## Introduction

Low back pain (LBP) is one of the common first presentations in primary care consultations and remains a major cause of disability worldwide. In the United Kingdom, LBP accounts for nearly 7.5% of all general practice consultations and represents one of the most frequent musculoskeletal complaints seen in primary care [1]. According to the World Health Organisation, an estimated 619 million people were affected by LBP in 2020, and this number is projected to rise to 843 million by 2050 [2]. Effective management of LBP can reduce the risk of chronic pain and long-term disability, minimise healthcare costs, and prevent repeated consultations in general practice. As LBP can significantly impact activities of daily living, early and appropriate intervention is crucial to maintaining function and quality of life [3-5]. Most cases of LBP are non-specific and self-limiting, yet they account for a substantial proportion of healthcare resource utilisation, absence from work, and socioeconomic burden [3,6].

The National Institute for Health and Care Excellence (NICE) provides evidence-based guidelines for the assessment and management of LBP: "Low back pain and sciatica in over 16s: assessment and management" [7]. The recommendations emphasise the early identification of red-flag symptoms, avoidance of unnecessary imaging, encouragement of self-management strategies, and the appropriate use of both pharmacological and non-pharmacological therapies. Adherence to these guidelines is essential to improve clinical outcomes, ensure cost-effectiveness, and provide patient-centred care [8]. Poor adherence to guideline recommendations has been associated with increased chronicity, unnecessary imaging requests, and inappropriate pharmacological prescribing, which may increase healthcare costs and worsen long-term outcomes [9,10].

Despite the existence of clear guidance, several studies have shown variation in the management of LBP within primary care. Reported deviations include the use of inappropriate pharmacological therapy and insufficient emphasis on self-management strategies [11,12]. As general practitioners play a pivotal role in shaping patient beliefs and expectations regarding LBP within primary care, inconsistencies in management can negatively affect recovery and contribute to chronicity [13]. Previous UK-based audits have reported significant variation in adherence to LBP management standards, indicating the need for ongoing local evaluation to ensure consistency with national guidance [14].

Regular audit and feedback cycles are therefore vital to ensure that practice remains aligned with national guideline recommendations and to identify areas requiring targeted improvement.

This audit aimed to evaluate adherence to the NICE guidelines in the management of first-presentation LBP within a single general practice in Glasgow, Scotland, and to identify areas for improvement.

## Materials and methods

Research design

The study was a retrospective cohort study aimed at evaluating adherence to the NICE guidelines titled "Low back pain and sciatica in over 16s: assessment and management" [7]. The audit assessed current practice in the management of the first presentation of LBP in a general practice setting. The current practice was compared with established guideline standards.

Study setting and duration

The study was conducted at a single general practice setting located in Glasgow, Scotland, serving a diverse patient population of approximately 3,300 individuals. The data obtained were between January 2024 and February 2025. The practice provides primary consultations and routine management for musculoskeletal conditions, including LBP.

Study subjects

The study sample comprised 71 patients who presented to the general practice with a first presentation of LBP. The data were obtained from electronic medical records using Read codes for patients presenting between January 2024 and February 2025.

A computer-generated random-number function in Microsoft Excel (Microsoft Corporation, Redmond, Washington) was used to select a sample of 71 patients. Randomisation was employed to minimise investigator selection bias and enhance the representativeness of the sample, ensuring that findings accurately reflected the diversity of patients, presentation, and management approaches within the general practice population. While randomisation was not used for inferential comparisons, it was applied to promote methodological transparency and reduce unconscious bias in patient selection, thereby strengthening the reliability of audit-based findings.

Inclusion and exclusion criteria

Inclusion criteria consisted of individuals aged 16 years and above presenting with their first presentation of LBP and no red-flag symptoms. The age limit of 16 years was selected in accordance with the NICE guideline "Low back pain and sciatica in over 16s: assessment and management" [7]. This age threshold aligns with the UK healthcare framework, which generally considers individuals aged 16 and above capable of providing informed consent for medical care. While some international guidelines use 18 years as the adult threshold, this audit followed the NICE criteria to ensure methodological consistency and relevance to UK primary healthcare practice.

Patients with a documented previous history of LBP, red-flag features (such as malignancy, infection, trauma, or neurological deficit), or those under 16 years of age were excluded.

Data collection

Data were extracted from electronic medical records, including clinical notes, prescription records, and referral letters. A sample of 71 patients was selected for participation in the audit. To minimise selection bias and ensure representative sampling, a computer-generated randomisation method was applied using Microsoft Excel. Each eligible patient identified through Read codes was assigned a unique number, and the Excel "RAND()" function was used to randomly select 71 patients from the list. This random selection process ensured that the sample reflected the general practice population presenting with first-episode LBP, therefore improving the reliability of findings.

Key metrics included advice on self-management, analgesic prescriptions, referral to physiotherapy, sick notes for work, and requests for imaging. All identifiable patient information was removed before data collection to maintain confidentiality.

A structured pro forma for data collection was developed based on the NICE guideline, "Low back pain and sciatica in over 16s: assessment and management" [7]. Each case was assessed for compliance with these guidelines. The data collection proforma included the parameters listed in Table 1.

**Table 1:** Key recommendations from the National Institute for Health and Care Excellence (NICE) guideline: "Low back pain and sciatica in over 16s: assessment and management" Source: NICE recommendation [7]

Guideline Domain	NICE Recommendation	Audit Parameter Assessed
Education and self-management advice	Provide reassurance and advice on remaining active, weight management, and self-management strategies. Discourage bed rest	Documentation of education and self-management advice
Pharmacological management	Offer NSAIDs as first-line treatment where appropriate; avoid routine opioid use for non-specific low back pain	Documentation of prescribed NSAIDs and opioid medications
Non-pharmacological management	Encourage regular exercise, physical activity, and physiotherapy referral or self-referral	Documentation of physiotherapy referrals/self-referrals
Work and function	Encourage continuation of normal activities and early return to work	Documentation of fit notes for work absence

Data analysis

Data were recorded and analysed using Microsoft Excel, with patient information anonymised. Each patient record was categorised as either "compliant" or "non-compliant" with the NICE guidelines.

Results were expressed as percentages to evaluate adherence to the NICE guidelines. Percentages were calculated by dividing the number of compliant cases by the total number of patients and multiplying this by 100. The findings were presented in a table to display adherence rates.

Ethical considerations

As this project was classified as a retrospective audit and service evaluation, formal ethical approval was not required according to local institutional policy. All data were anonymised before analysis, and no identifiable patient information was collected, stored, or reported.

Quality improvement interventions

Following data analysis, the findings of the first audit cycle were presented at a local practice meeting. An educational poster summarising the current NICE guidelines was displayed within the practice to promote staff awareness. These interventions were conducted to standardise practice and improve compliance with evidence-based management of LBP.

Audit cycle

This study represented the first cycle of a quality improvement process. A re-audit is planned following the implementation of the educational interventions to assess the effectiveness of these strategies and ensure ongoing improvement in patient care and adherence to NICE guidelines.

## Results

A total of 71 patient cases with a first presentation of LBP were reviewed. Of these, 39.4% (n = 28) received management that was fully compliant with the NICE guideline "Low back pain and sciatica in over 16s: assessment and management" [7], while 60.6% (n = 43) did not meet the guideline standards.

Education and self-management

Patient education was provided in 39.4% (n = 28) of cases, consistent with NICE recommendations. Exercise was the most frequently advised strategy (36.6%, n = 26), followed by weight loss (8.5%, n = 6) and other lifestyle recommendations such as yoga, pilates, or massage therapy (4.2%, n = 3). The remaining 60.6% (n = 43) had no documentation of education or self-management advice consistent with NICE guidelines. This represents a key area for improvement.

Pharmacological management

Non-steroidal anti-inflammatory drugs (NSAIDs) were prescribed to 21.1% (n = 15) of cases, consistent with NICE guidelines as the first-line pharmacological treatment. Opioid analgesia was prescribed to 9.9% (n = 7) of cases, which deviates from the NICE guidance. Routine opioid use is discouraged for non-specific LBP.

Non-pharmacological management

Physiotherapy self-referral was documented for 39.4% (n = 28) of patients, demonstrating good adherence to guideline standards encouraging physical activity and exercise-based therapy.

Work and function

Fit notes for work absence were issued in 4.2% (n = 3) of cases. NICE guidelines recommend maintaining normal activity where possible; therefore, this reflects minimal reliance on work absence as a management strategy.

Overall guideline compliance

Overall, 39.4% (n = 28) of patients were fully compliant across all domains of NICE guideline recommendations. Table 2 summarises the audit findings across five management domains.

**Table 2:** Adherence to the National Institute for Health and Care Excellence​ (NICE)​ guidelines for the management of first-presentation low back pain Source: Management domain [7]

Management Domain	Intervention	Number of Patients (n)	% of Cases Compliant to Guidelines
Education and self-management	Provision of patient education or self-management advice	28	39.4%
Pharmacological management	Non-steroidal anti-inflammatory drugs (NSAIDs) prescription	15	21.1%
Pharmacological management	Opioid prescription	7	9.9%
Non-pharmacological management	Physiotherapy self-referral	28	39.4%
Work and function	Sick notes for work absence	3	4.2%
Overall guideline compliance	Fully compliant management across all domains	28	39.4%

Implementation of quality improvement measures

**Figure 1:**
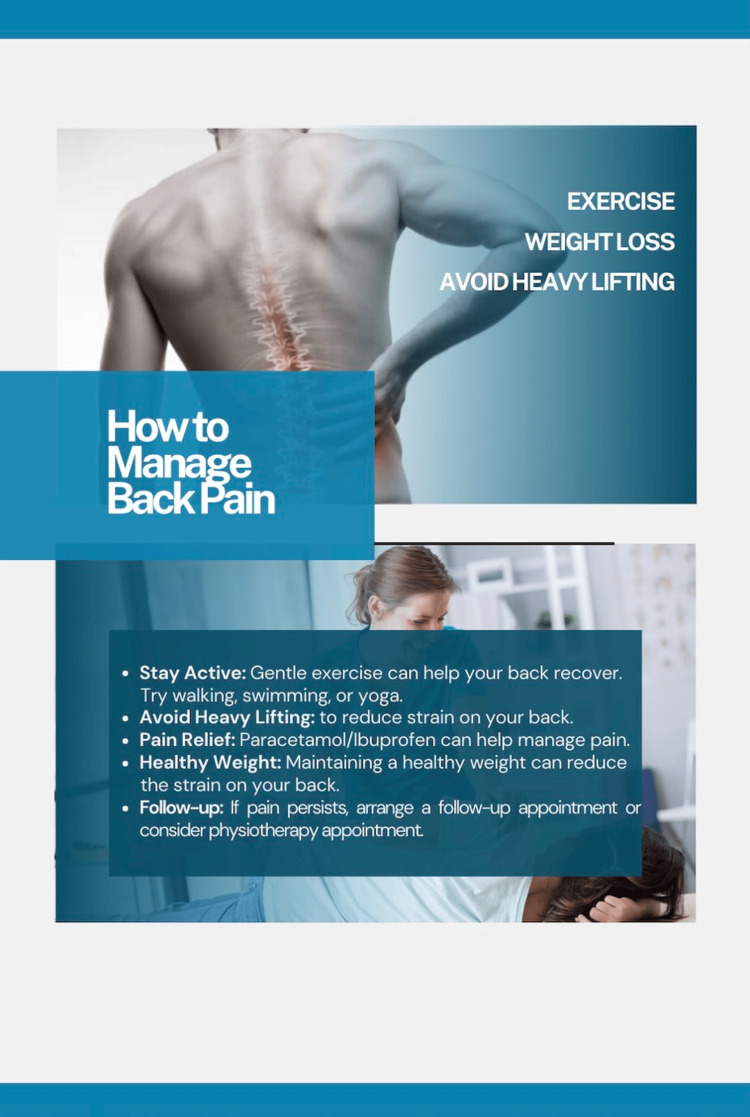
Educational poster displayed within the practice. Source: Created by the authors based on the NICE guideline recommendations (2020) "Low back pain and sciatica in over 16s: assessment and management" [7]

Following completion of the audit, an educational poster summarising the NICE guidelines was created and displayed within the practice to raise staff awareness and support adherence to evidence-based management (Figure 1).

## Discussion

This audit evaluated adherence to the NICE guideline "Low back pain and sciatica in over 16s: assessment and management" [7] for the management of first-presentation LBP within a primary care setting. The findings highlight both areas of strength and opportunities for targeted improvement in current clinical practice. Similar trends have been reported in UK primary care audits, indicating persistent variation in adherence to musculoskeletal management guidelines across NHS settings [2,14].

Overall, only 39.4% (n = 28) of patients received management advice that was fully compliant with the NICE guideline recommendations [7]. The most significant gap identified was patient education and self-management advice. Education was provided in only 39.4% of cases, despite strong evidence demonstrating its importance in improving pain outcomes, promoting functional recovery, and reducing long-term disability [12,13,15,16]. Most patients (60.6%) did not receive documented education or advice in line with NICE recommendations, which emphasise reassurance, maintenance of activity, and discouragement of bed rest [7]. Previous studies have reported similar findings, highlighting that patient education remains underutilised in primary care and is often inconsistently documented [8,15,17,18]. This suggests a continued gap between evidence-based guidance and clinical implementation. Education has been shown to reduce fear-avoidant behaviour and enhance self-efficacy in managing LBP, thereby supporting long-term recovery and preventing chronicity [11,16,19].

With regard to pharmacological management, NSAIDs were prescribed appropriately in 21.1% (n = 15) of cases, aligning with NICE guidance as first-line pharmacological therapy [7]. However, 9.9% (n = 7) of patients were prescribed opioid analgesia, which is discouraged in the NICE guideline due to limited efficacy and risk of harm [7,20]. The overprescription of opioids for non-specific LBP has been similarly observed in primary care studies worldwide, despite robust evidence highlighting the risks of dependency and lack of long-term benefit [13,20,21]. Non-opioid pharmacological options, alongside education and exercise-based therapy, have demonstrated superior outcomes in both pain management and functional improvement [20,21]. These findings highlight a key area where clinician education could strengthen adherence to evidence-based pharmacological care.

Non-pharmacological interventions were more consistently followed. Physiotherapy self-referral was documented in 39.4% (n = 28) of cases, which aligns with NICE guidance promoting early engagement in physical activity and exercise-based rehabilitation [7]. Exercise-based interventions have consistently been shown to improve pain, physical function, and quality of life in patients with LBP [17,22-24]. Similarly, only a small proportion of patients (4.2%, n = 3) received fit notes for work absence, which is consistent with guideline recommendations encouraging continued activity where possible. Maintaining work engagement has been linked to faster recovery and reduced disability risk in patients with LBP [18,23].

The audit results are consistent with previous UK-based studies, which have also identified variability in adherence to LBP management guidelines in primary care [15,17,19]. Regular audit and feedback interventions have been shown to significantly improve professional practice and clinical outcomes, particularly when findings are communicated directly to the clinical team [25,26]. This reinforces the need for ongoing local audits and structured feedback to ensure continuous quality improvement. In this practice, an educational poster summarising NICE recommendations was introduced, and findings were presented at a departmental meeting to promote awareness and standardise care. A re-audit is planned to assess the effectiveness of these interventions in improving compliance and patient outcomes. Including audit cycles as part of continuous professional development has been recognised as an effective mechanism to maintain evidence-based practice and enhance long-term adherence to national guidelines [27]. Embedding NICE guideline prompts within electronic medical record systems and promoting clinician education could further enhance adherence to evidence-based management in primary care [24]. This audit represents the first cycle of a quality improvement process aimed at enhancing adherence to NICE guidelines.

Strengths and limitations

Overall, these findings demonstrate measurable areas for practice improvement and emphasise the value of continuous audit in ensuring adherence to national guidelines. Strengths of this audit include a well-defined patient cohort, structured data collection aligned with national guidelines, and a direct link between findings and quality improvement actions. However, limitations should be acknowledged. The single-centre design limits generalisability, and the sample size was modest. Documentation bias may also have underestimated the true rate of patient education provided verbally but not recorded in the notes. Additionally, the retrospective nature of the audit precludes assessment of long-term outcomes or patient adherence following the consultation.

Implications for practice

The findings underscore the importance of integrating education and self-management advice into routine consultations for patients presenting with LBP. Future initiatives should focus on clinician education, improved documentation, and embedding guideline-based prompts within electronic medical records. Ongoing audit cycles will be essential to sustain adherence and measure the impact of these quality improvement measures.

## Conclusions

This audit demonstrated key areas of non-adherence to the NICE guideline for the management of first-presentation LBP within a primary care setting. While pharmacological and physiotherapy management were generally appropriate, significant gaps were observed in the provision and documentation of patient education and self-management advice, which are core components of evidence-based care. These findings emphasise the need to strengthen awareness and implementation of guideline-based practice among clinicians to ensure patients receive consistent, evidence-based management of LBP.

In response to the audit findings, an educational poster summarising the NICE guideline recommendations was introduced, and results were presented within the practice to reinforce evidence-informed management. A re-audit is planned to evaluate the effectiveness of these interventions. Ongoing audit cycles and targeted clinician education are essential to sustain improvements in adherence and optimise patient outcomes in primary care. Incorporating these strategies across primary care settings may enhance the standardisation of LBP management nationally.

## References

[1] Maniadakis N, Gray A (2000). The economic burden of back pain in the UK. Pain.

[2] (2025). Low back pain. https://www.who.int/news-room/fact-sheets/detail/low-back-pain.

[3] Wu A, March L, Zheng X (2020). Global low back pain prevalence and years lived with disability from 1990 to 2017: estimates from the Global Burden of Disease Study 2017. Ann Transl Med.

[4] Hartvigsen J, Hancock MJ, Kongsted Kongsted (2018). What low back pain is and why we need to pay attention. Lancet.

[5] Artus M, van der Windt D, Jordan KP, Croft PR (2014). The clinical course of low back pain: a meta-analysis comparing outcomes in randomised clinical trials (RCTs) and observational studies. BMC Musculoskelet Disord.

[6] Fatoye F, Gebrye T, Ryan CG, Useh U, Mbada C (2023). Global and regional estimates of clinical and economic burden of low back pain in high-income countries: a systematic review and meta-analysis. Front Public Health.

[7] (2025). Low back pain and sciatica in over 16s: assessment and management. https://www.nice.org.uk/guidance/ng59.

[8] Williams CM, Maher CG, Hancock MJ (2010). Low back pain and best practice care: a survey of general practice physicians. Arch Intern Med.

[9] Pike A, Patey A, Lawrence R (2022). Barriers to following imaging guidelines for the treatment and management of patients with low-back pain in primary care: a qualitative assessment guided by the Theoretical Domains Framework. BMC Prim Care.

[10] Darlow B, Dowell A, Baxter GD, Mathieson F, Perry M, Dean S (2013). The enduring impact of what clinicians say to people with low back pain. Ann Fam Med.

[11] Traeger AC, Hübscher M, Henschke N, Moseley GL, Lee H, McAuley JH (2015). Effect of primary care-based education on reassurance in patients with acute low back pain: systematic review and meta-analysis. JAMA Intern Med.

[12] Qaseem A, Wilt TJ, McLean RM (2017). Noninvasive treatments for acute, subacute, and chronic low back pain: a clinical practice guideline from the American College of Physicians. Ann Intern Med.

[13] Koes BW, van Tulder M, Lin CW, Macedo LG, McAuley J, Maher C (2010). An updated overview of clinical guidelines for the management of non-specific low back pain in primary care. Eur Spine J.

[14] Duarte ST, Moniz A, Costa D, Donato H, Heleno B, Aguiar P, Cruz EB (2024). Low back pain management in primary healthcare: findings from a scoping review on models of care. BMJ Open.

[15] Liddle SD, Baxter GD, Gracey JH (2004). Exercise and chronic low back pain: what works?. Pain.

[16] Scott NA, Moga C, Harstall C (2010). Managing low back pain in the primary care setting: the know-do gap. Pain Res Manag.

[17] Foster NE, Anema JR, Cherkin D (2018). Prevention and treatment of low back pain: evidence, challenges, and promising directions. Lancet.

[18] Buchbinder R, Tulder MV, Oberg B (2018). Low back pain: a call for action. Lancet.

[19] Chou R, Turner JA, Devine EB (2015). The effectiveness and risks of long-term opioid therapy for chronic pain: a systematic review for a National Institutes of Health Pathways to Prevention Workshop. Ann Intern Med.

[20] Krebs EE, Gravely A, Nugent S (2018). Effect of opioid vs nonopioid medications on pain-related function in patients with chronic back pain or hip or knee osteoarthritis pain: the SPACE randomized clinical trial. JAMA.

[21] Hayden JA, van Tulder MW, Malmivaara A, Koes BW (2005). Exercise therapy for treatment of non-specific low back pain. Cochrane Database Syst Rev.

[22] van Tulder M, Malmivaara A, Esmail R, Koes B (2000). Exercise therapy for low back pain: a systematic review within the framework of the Cochrane Collaboration Back Review Group. Spine (Phila Pa 1976).

[23] Maher C, Underwood M, Buchbinder R (2017). Non-specific low back pain. Lancet.

[24] Wilson R, Pryymachenko Y, Abbott JH (2023). A guideline-implementation intervention to improve the management of low back pain in primary care: a difference-in-difference-in-differences analysis. Appl Health Econ Health Policy.

[25] Jamtvedt G, Flottorp S, Ivers N (2019). Audit and feedback as a quality strategy. Improving Healthcare Quality in Europe: Characteristics, Effectiveness and Implementation of Different Strategies.

[26] (2025). Principles for best practice in clinical audit. https://www.nice.org.uk/media/default/About/what-we-do/Into-practice/principles-for-best-practice-in-clinical-audit.pdf.

[27] Glenngård AH, Anell A (2021). The impact of audit and feedback to support change behaviour in healthcare organisations - a cross-sectional qualitative study of primary care centre managers. BMC Health Serv Res.

